# Multicultural identity integration and well-being: a qualitative exploration of variations in narrative coherence and multicultural identification

**DOI:** 10.3389/fpsyg.2013.00126

**Published:** 2013-03-14

**Authors:** Maya A. Yampolsky, Catherine E. Amiot, Roxane de la Sablonnière

**Affiliations:** ^1^Département de Psychologie, Université du Québec à MontréalMontreal, QC, Canada; ^2^Département de Psychologie, Université de MontréalMontreal, QC, Canada

**Keywords:** multicultural identity, identity integration, well-being, narrative coherence, life story

## Abstract

Understanding the experiences of multicultural individuals is vital in our diverse populations. Multicultural people often need to navigate the different norms and values associated with their multiple cultural identities. Recent research on multicultural identification has focused on how individuals with multiple cultural groups manage these different identities within the self, and how this process predicts well-being. The current study built on this research by using a qualitative method to examine the process of configuring one's identities within the self. The present study employed three of the four different multiple identity configurations in Amiot et al. (2007) cognitive-developmental model of social identity integration: categorization, where people identify with one of their cultural groups over others; compartmentalization, where individuals maintain multiple, separate identities within themselves; and integration, where people link their multiple cultural identities. Life narratives were used to investigate the relationship between each of these configurations and well-being, as indicated by narrative coherence. It was expected that individuals with integrated cultural identities would report greater narrative coherence than individuals who compartmentalized and categorized their cultural identities. For all twenty-two participants, identity integration was significantly and positively related to narrative coherence, while compartmentalization was significantly and negatively related to narrative coherence. ANOVAs revealed that integrated and categorized participants reported significantly greater narrative coherence than compartmentalized participants. These findings are discussed in light of previous research on multicultural identity integration.

## Introduction

As our societies become more culturally complex, it is vital that we understand how individuals deal with diverse cultural contexts. More and more people are in a position where they need to negotiate the different cultural identities that are derived from their own belonging to different cultural groups (Mahtani, 2002; Giguère et al., 2010). People who belong to more than one cultural group must navigate the diverse norms and values from each of their cultural affiliations. Faced with such diversity, multicultural individuals need to manage and organize their different and possibly clashing cultural identities within their general sense of self. An emerging body of research accounts for how multicultural individuals reconcile and organize their different cultural identities within themselves (e.g., Haritatos and Benet-Martínez, 2002), and how this process is related to well-being (e.g., de la Sablonnière et al., 2010). This research suggests that the manner in which one's diverse cultural identities are integrated and organized predicts well-being. At the same time, a more profound account of this complex identification process and its relation to well-being is warranted. The primary objective of the present research is to gain deeper insight into this multicultural identification process by first, qualitatively investigating the identity experiences of multicultural individuals, and second, by investigating several patterns or configurations for identifying with multiple cultural groups. This was achieved by using a cognitive-developmental model developed by (Amiot et al., 2007). Specifically, the present research uses a narrative approach to investigate how different identification patterns or configurations are related to individuals' well-being, indicated by how coherently people told their life story.

### Multicultural societies and individuals

The diversity of our North American society has increased as a result of migration and globalization (Sam and Berry, 2006). In Canada, Jantzen (2009) reported that less than 30% of citizens belonging to the second generation are from British or French Canadian origin only, while over 30% are exclusively from European countries, and approximately 20% is comprised of non-European peoples (e.g., South or East Asians, Latin Americans, Natives, etc.). Reports from the 2010 US census (Humes et al., 2011) show that ethnic minorities—non-Caucasians—form 18% of the country's population. These statistics attest to the important multiplicity of North American citizens. The present study was conducted in Montreal, which is one such city that reflects this linguistic and cultural diversity. Montreal has two official mainstream languages: English and French. In terms of these mainstream languages, approximately 31% of the city's inhabitants are Francophone (French-speakers), 10% are Anglophone (English-speakers), while approximately half of the population of Montreal is English–French bilingual. In addition to the mainstream languages, 20% of the city's population speaks a non-official language at home (Statistics Canada, 2012). In terms of cultural and ethnic diversity, approximately 16% are from non-European ethnic groups (Statistics Canada, 2012), including South and East Asian, Latin American, Middle-Eastern, Caribbean, African, and “mixed” heritage groups.

The complex “superdiversity” (see Vertovec, 2007) that has emerged in several countries and cities—including Montreal—is qualified by a convergence of innumerable national, religious, language and ethnic groups in the same environment. In addition, the contact between these multiple cultural groups over time produces boundary-blurring, hybrid experiences (Pieterse, 2001). Such complexity has implications for the subjective experiences of the individuals who belong to multiple cultural groups; these implications include their ability to integrate the threads of their diverse cultural experiences into a coherent, unified understanding of themselves and their lives. For multicultural individuals, including first generation immigrants, descendants from first generation immigrants, and people with a “mixed” cultural heritage, living in more than one cultural group requires them to negotiate and integrate differing expectations, norms, values, and practices associated with their multiple cultural identities (Giguère et al., 2010).

### Multicultural identity integration

There has been a rise in the number of approaches for understanding the experience of multicultural individuals. The acculturation literature has investigated biculturals and how they reconcile their belonging to different cultural groups (see Ryder et al., 2000; Sam and Berry, 2006). Acculturation research has conventionally employed Berry's bidimensional model (e.g., Berry and Sam, 1997); this model proposes four acculturation orientations: exclusive belonging to either the heritage or mainstream cultural group, belonging to both heritage and mainstream groups (i.e., integration orientation), or belonging to neither heritage nor mainstream groups. Whereas the acculturation research typically focuses on membership and involvement in the heritage and mainstream cultural groups, recent work has begun to focus more deeply on how biculturals and multiculturals intra-individually integrate their different cultural identities within themselves, and how they subjectively reconcile these different identities (e.g., Haritatos and Benet-Martínez, 2002). For example, Benet-Martínez and colleagues' seminal work on bicultural identity integration (BII; e.g., Benet-Martínez et al., 2002; Haritatos and Benet-Martínez, 2002) looks at whether or not the relationship between one's cultural identities is compatible through the perception of harmony and similarity, vs. incompatible through the perception of conflict and distance. The work of Noels et al. (1996) highlighted how bicultural people will “frame switch,” or shift from one set of behaviors to another, depending on the context, as a way to manage these different cultural identities. Kawakami et al. (2011) also discuss this intra-individual adaptation to one's different contexts; their work shows that contexts activate different social categories, which predict the synchronization of one's self-concept to the salient category. Downie et al. (2004, 2006) propose that having compatible cultural identities involves feeling consistent across identities and contexts, rather than having distinct and fragmented cultural identities that shift from one context to another (i.e., cultural chameleonism). Tadmor and Tetlock (2006) account for the intra-individual negotiation of one's alignment with more than one cultural group, and show that multiple alignments can lead to greater bicultural integrative complexity, which involves perceiving one's differing cultural perspectives as equally valid and advantageous, particularly in the resolution of conflicts (see also Tadmor et al., 2009). In the developmental literature, several scholars have highlighted the dynamic nature of cultural identification, and how it is subject to change over different phases of life as one reconciles ones multiple roles (see Phinney, 1989; Harter and Monsour, 1992; Harter and Whitesell, 2003). Each of these models highlight important components of the multicultural identification process, and they each capture how multicultural individuals intra-individually organize their diverse cultural identities, which also has important repercussions for individual well-being. Building on this prior work, the present study uses the framework of Amiot et al. (2007) cognitive-developmental model, which integrates many of these different theories into a single model and focuses on the processes of identity integration, described below.

### The cognitive-developmental model of social identity integration and well-being

In order to examine different configurations for identifying with one's multiple cultural groups and capture the dynamic nature of these identification patterns, we employ the cognitive-developmental model of social identity integration (CDMSII; Amiot et al., 2007). This model builds on previous work on bicultural identification and development (e.g., Phinney and Devich-Navarro, 1997; Haritatos and Benet-Martínez, 2002) and accounts for a broad range of identification configurations. The main strength of the CDSMII is its detailed account of how multiculturals cognitively combine their many cultural identities within the self, and how these cognitive configurations can change over time and across life events.

The CDMSII proposes different ways that people can cognitively configure their different cultural identities within their self-concept. There are four different intra-individual configurations proposed by the model: anticipatory categorization, categorization, compartmentalization, and integration. Anticipatory categorization takes place while one is preparing to become a member of a new group by projecting oneself into their future cultural group, and by foreseeing similarities between oneself and the future group. However, since the current study examines the experiences of multiculturals who have already grown up with multiple cultural groups, we focus on the categorization, compartmentalization, and integration configurations.

Categorization is characterized by the dominance of a single cultural identity over others in defining the self, and the person identifies with one culture to the exclusion of others. For example, a second-generation Canadian person of Ukrainian heritage may identify predominantly as Canadian, while dis-identifying with his Ukrainian heritage culture. Identifying with one culture can be seen as a way to simplify one's identification experience (e.g., Roccas and Brewer, 2002).

Compartmentalization involves identifying with one's multiple cultural groups, but these identities are perceived as fundamentally disparate and are kept separate from each other. As such, a person with compartmentalized identities may feel that the differences between her identities are irreconcilable, and see them as completely independent parts of her self-concept. Moreover, identification remains bound to the surrounding context, such that one identifies with their cultures depending on the context. To illustrate, one may identify as both Indian and Canadian, but this person will only identify as Indian when she is with other Indians, and with Canadians when with other Canadians; the two identities are rarely experienced at the same point in time. By compartmentalizing the identities as separate entities within the self, one is able to avoid any conflict that could occur if identities are perceived as contradictory.

Integration involves reconciling and connecting one's diverse cultural identities. This is achieved by perceiving similarities between these different identities. In addition, the differences between the identities are recognized, but are deemed to complement and enrich each other rather than to clash. For example, one can identify as Chinese, Iranian, and Canadian, and see that there are many shared values between his three identities, and that the differences between these cultural identities (e.g., in terms of norms or values) provide him with diverse perspectives. The experience of simultaneous identification also characterizes the integration configuration, so that while people adapt as they move from one cultural context to another, they still feel that they belong to all their cultural groups. For example, a multicultural person who has integrated his different cultural identities may attend Sufi rituals with his Iranian–Canadian father, refer to Steven Chow films with his Cantonese–Chinese–Canadian cousins, and speak English and French with friends at school and work; yet as this person engages in each of these cultural activities, he sees that each of his cultural identities are connected, and feels that he is part of all of his cultural groups, simultaneously. A superordinate identity—a larger, overarching identity category that includes one's different cultural identities (e.g., being human)—may also be invoked to reconcile and link one's different cultural identities together (see Gaertner et al., 1993). The CDSMII also accounts for the variability that exists in these patterns of cultural identification over time; in drawing on developmental principles (e.g., Harter and Monsour, 1992; Harter, 1999), the model states that one would likely move from categorization, to compartmentalization, to integration as one internalizes and reconciles their different identities over time.

The CDSMII is useful for the study of multicultural individuals' identity configuration experience for several reasons. First, it accounts for different ways in which multicultural individuals may identify with their heritage and mainstream groups, and also examines the interdependence and the negotiation between these identities (e.g., Rudmin, 2003, 2006). The model seeks to account for the flexible nature of cultural identification and for the possibility that the identity configurations change over time. Moreover, this model integrates and elaborates on previous work in cultural identity integration. Relative to Benet-Martínez's BII model (e.g., Benet-Martínez and Haritatos, 2005), the integration configuration of the CDMSII involves a subjective feeling that one's different identities are similar and interrelated, and therefore accounts for the similarity component of the BII. The CDMSII emphasizes additional cognitive strategies involved in identity integration, such as recognizing that one's different cultural identities possess differences that complement each other, and identifying with a superordinate category. The integration configuration of the CDMSII also includes the experience of reconciling the discrepancies between one's different identities. This overlaps with Tadmor and Tetlock's (2006; Tadmor et al., 2009) model of acculturative complexity, which involves facing and coping with cultural conflict by negotiating the differences between one's different cultural affiliations as one comes to identify with each group. As identity integration involves a sense of simultaneous identification with one's different cultural groups, this construct is comparable to the subjective feeling of consistency experienced across different cultural contexts purported by Downie et al. model of cultural chameleonism vs. integration (2006, 2004). The compartmentalization configuration of the CDSMII involves perceiving one's cultural identities as divergent and separate from one another, a characteristic that also represents the distance dimension proposed in the BII; compartmentalization also ties well with the context-bound identification experience noted by Downie et al. (2006, 2004). The categorized configuration of the CDSMII includes the experience of siding with one cultural group over another. This phenomenon has similarly been described in Tadmor and Tetlock's (2006) acculturation complexity model; in their model, the authors propose that as a person moves through their bicultural experiences and feels accountable to a single cultural group, rather than to multiple groups, then one would be more likely to choose either an assimilation or separation strategy, and identify accordingly. In the same vein, categorization ties to the assimilation and separation acculturation strategies discussed in Berry and Sam (1997) acculturation model, and in the work of Phinney and Devich-Navarro (1997).

Because of its comprehensive nature, the CDSMII was chosen as a guide for understanding the identity experiences of multicultural individuals, and as a structure for the rich, qualitative approach used in the present research. The identity configurations proposed in the model are employed in the present study because of their expected links with well-being.

### Multicultural identity integration and well-being

The manner in which one's different cultural affiliations are negotiated consistently predicts individual well-being. The literature examining the well-being of biculturals and multiculturals suggests that integrating one's cultural identities—being involved in both one's mainstream and one's heritage cultural groups—seems to yield greater well-being. In the acculturation research, Berry et al. (2006) revealed that immigrant adolescents who reported an integration orientation also experienced greater life satisfaction, self-esteem, and less behavioral problems compared to those who only associated with their heritage culture, those who only assimilated into the majority culture, and those who were marginalized outside of both heritage and majority cultures. A recent meta-analysis of the acculturation literature by Nguyen and Benet-Martínez (2013) demonstrated that biculturalism, or affiliating with both heritage and mainstream cultural groups, was significantly and positively associated with psychological adjustment, more so than associating solely with either the heritage or the mainstream.

These findings from the acculturation research indicate that being involved in both heritage and mainstream cultural groups predicts enhanced well-being, but of greater interest in the present study is how well-being is predicted by the intra-individual process of negotiating and combining one's different identities. Evidence that multicultural identity integration is associated with individual well-being is gradually emerging. Downie et al. (2004) found that bicultural individuals' perception of intra-individual consistency between their cultural identities, as opposed to context-bound identification, predicted greater psychological well-being. In their review of the literature surrounding social identity integration, Cheng et al. (2008) found that those who perceived their identities to be compatible were more likely to report greater well-being outcomes. In the domain of intergroup relations, priming one's multiple group affiliations leads to greater resilience than priming a single group affiliation (Jones and Jetten, 2010).

In line with this work, the CDMSII also proposes that identity integration should predict greater well-being as it involves reconciling one's identities in order to achieve greater coherence within oneself (Amiot et al., 2007). A few studies have begun to examine the relationship between the CDMSII's identity integration and well-being. Developing a sense of identification with a new social group predicts increased vitality and well-being (Amiot et al., 2010). In the area of cultural identities specifically, the work of de la Sablonnière et al. (2010) demonstrated that identifying equally with one's different cultures was a significant predictor of self-esteem, vitality, life satisfaction and overall well-being. Immigrants who identify with all of their cultural groups (mainstream and heritage) reported greater collective hope and well-being, self-esteem, life satisfaction, and vitality than those who categorized their cultural identities (Carpentier and de la Sablonnière, 2013).

Thus far, the research on identity integration and well-being indicates that integrating one's multiple cultural identities predicts greater well-being than compartmentalizing or categorizing the identities. The present research seeks to deepen this line of inquiry by first qualitatively capturing the complexity of the multicultural identity configuration experience using the CDSMII. Second, the research employs a narrative approach to study the link between these identity configurations and the outcome of well-being. Very little research has employed narratives to consider the experiences of multiculturals (e.g., Bougie et al., 2011). The use of narratives, and narrative coherence as the well-being indicator, is a rich and novel component in our account of multicultural individuals' well-being experiences. Using the life narrative approach was consequently considered a method of choice to capture the richness of multicultural individuals' identity configurations and their well-being.

### Narrative coherence as a relevant well-being indicator among multicultural individuals

While emerging research provides some support for the relationship between identity integration and well-being, the findings thus far are limited to quantitative approaches. We use a qualitative approach in the present research to harness the richness of multicultural individuals' identity experiences, while also deriving their well-being through the narration of their own cultural life stories. A life story can provide profound insight into an individual's well-being, since narratives construct and reveal values and identity, personal meaning, and sense of continuity and coherence over time (McAdams, 1985). Chandler and Lalonde (1999), Chandler et al. (2003), and Chandler and Proulx (2006) influential work has also accounted for the importance of personal and cultural continuity; more specifically, they have examined how one's understanding and expression of their experiences of both change and consistency over the course of their own life stories relates to the critical outcome of suicide, specifically for Native-Canadian populations. Harter et al. (1997) demonstrated that one's life story can reveal how one moves from experiencing role conflict within one's self-portrait in adolescence, to reconciling one's multifaceted self in adulthood. Coherence in one's narrative represents one's ability to effectively make sense of one's life and to be understood, and it relates to living well (Colby and Damon, 1992; Rosenwald and Ochberg, 1992). Narrative coherence has also been used as an indicator of well-being in both social psychology and clinical research (see Baerger and McAdams, 1999; Adler et al., 2007). Furthermore, the narrative method has been conducted with multicultural groups, such as Inuit in Canada (de la Sablonnière et al., 2011), and Anglophone and Francophone Quebecers (Bougie et al., 2011). The indicator of narrative coherence is useful and relevant for understanding multiculturals' well-being since these individuals go through a process of reconciling their different identities within themselves, and the narratives enable us to identify how well they are faring in this process of making sense of themselves vis-à-vis their diverse and potentially conflicting cultural affiliations.

In order to render the broad concept of narrative coherence more tangible, it is necessary to capture its different components or processes by which the person makes sense of their life events. The four dimensions of narrative coherence, delineated by Baerger and McAdams (1999) include: orientation, structure, affect, and integration. Orientation is the process of setting the stage for the audience by providing contextual parameters such as time, location, characters, and other personal circumstances. This is an important first dimension of coherence as it locates the events of one's story in time and place, rather than leaving the narrated events floating in space. Proficient orientation reveals the participant's awareness of the context for the events that transpired in their own life story (Peterson and McCabe, 1983; Baerger and McAdams, 1999). Structure designates how well the story follows the logical progression of an episode, where one describes an initiating event, followed by a personal response to this event (e.g., an evaluation, a desire to resolve an issue, a goal), followed by a pursuit in accordance with one's response (e.g., attempts to solve challenges or achieve goals) and the subsequent consequences of these events and reactions. Structure is essential to the organization and clarity of one's life events, and demonstrates how well the participant can frame their experiences in time, rather than bouncing from event to event with no apparent purpose or connection to them (McAdams, 1993). Affect is the emotional or evaluative tone of the story, where the participant expresses positive or negative evaluations of their circumstances, activities and reactions. Affect shows how the participant positions oneself vis-à-vis their life events, and how they feel about their experiences, rather than simply listing events with no personal input (McAdams, 1996). As a result, greater presence of affective content contributes positively to the coherence of one's narrative. Lastly, integration denotes the linking of one's circumstances, events, and responses to larger, overarching themes of one's complete narrative. In addition, episodic inconsistencies and unsettled issues are reconciled with each other. Integration demonstrates how well the participant can understand and process their experiences, and derive meaning from their life (McAdams, 1993, 2001).

Baerger and McAdams found that these components of narrative coherence were significantly related to measures of well-being. Namely, narrative coherence demonstrated a moderate positive correlation with measures of happiness and satisfaction with life, and a significant negative correlation with depression. Furthermore, incoherence in one's narratives has been shown to potentially denote psychopathology, such as schizophrenia (see Roe, 2001; Lysaker and Lysaker, 2002). These findings demonstrate that coherence is not only closely related to well-being, but can also be seen as a well-being indicator in and of itself. The present research applied the narrative method to investigate the relationship between the different cultural identity configurations and well-being among multicultural individuals. We considered coherence to be an especially relevant indicator of well-being for this population because multicultural individuals live with multiple cultural meaning systems, each with their own varied set of values, norms, and practices to follow (Giguère et al., 2010). While there is indeed overlap between each cultural system, there is still a great deal of difference to reconcile and resolve as one finds where one fits within these structures (Stroink and Lalonde, 2009). These diverse identity experiences can be personally enriching (e.g., Tadmor et al., 2009), but they can also be confusing and stressful (e.g., Phinney and Devich-Navarro, 1997; Gupta, 1999; Lalonde and Giguère, 2008), and potentially threatening to one's mental health (e.g., Cantor-Graae and Pedersen, 2007; Bourque et al., 2011). Personal coherence may therefore be more challenging to achieve, but once attained represents a successful resolution of the diverse and complex cultural aspects of one's self.

### The present research

The current study aims to examine how multicultural individuals configure their diverse cultural identities using a qualitative approach, to verify the changes in one's cultural identity configurations over the course of the life narrative, and to test how these configurations are related to the coherence of their life narratives as an indicator of well-being. It was predicted that identity integration would be positively related to all four dimensions of narrative coherence: orientation, structure, affect and narrative integration. This is because the process of integrating one's multiple cultural identities involves resolving conflicts and finding connections between one's identities in order to achieve a more cohesive self-concept. We also predicted that compartmentalization would be related to lower narrative orientation, structure, affect, and integration than identity integration, since keeping each identity in separate compartments within oneself implies that one is avoiding conflicts between one's identities rather than synchronizing these different parts of oneself. Since multicultural individuals who align themselves exclusively with the mainstream or heritage culture do not fare as well as those who align with both (e.g., Sam and Berry, 2006; Nguyen and Benet-Martínez, 2013), it was expected that categorization would be associated with less coherence than compartmentalization on all four dimensions of narrative coherence.

## Methods

### Participants

For the present research, we focus on the population of second and higher generation descendants of immigrants as well as those with multiple cultural heritages (“mixed”). This is because these individuals have lived and have been socialized with more than one cultural group from childhood to the present; as a result they are familiar with being multicultural, and their multiple cultural affiliations are salient. In addition, the current research allowed for greater representation of this under-represented population. Twenty-two multicultural Canadians were recruited through the first author's network of distant contacts and through snowballing, where participants and friends referred individuals to the first author. Nine men and thirteen women participated in the study, with ages ranging from 18 to 26 years old (*M* = 23.23). All of the participants had received, or were in the process of pursuing, university degrees. Twelve participants were pursuing university studies at the time, eight were working full time, and two had not mentioned their current employment status.

The cultural backgrounds of the participants were diverse. Most were first generation Canadians: 18 were born in Canada, 2 moved to Canada as toddlers, one came to Canada while in elementary school, and one came from Mauritius for her university studies initially, and then stayed since to continue working. All were Canadian citizens at the time of the interviews, except for the participant from Mauritius, who was in the process of applying for resident status.

Fifteen participants were of mixed heritages, where their parents came from different cultural backgrounds. The other seven had parents with the same cultural background. All of the participants listed either the English or French Canadian/Quebecer mainstream groups as one of their cultural memberships. The heritage cultural affiliations represented in this sample include English and French Canadian/Quebecer, Inuk, American, Jewish, Mexican, Haitian, Italian, Irish, Scottish, Swedish, French, British, Indo-British, Indian, Bangladeshi, Lebanese, Syrian, Armenian, Moroccan, Filipina, Japanese, Chinese, Chinese-Mauritian, and Chinese–Guyanese.

### Procedure

Once written consent was obtained, participants were interviewed in English by the first author at the Université du Québec à Montréal; the duration of the interviews ranged from 40 to 126 min in length (*M* = 75.68 min, *SD* = 27.35 min). The interviews started with the participants' naming and briefly describing their cultural groups. Next, the interview was presented as consisting of two sections: a story section, and a section with specific questions about their identities. The first half of the interview structure always consisted of a narrative section. Narratives enabled participants to tap into their cultural experiences and to communicate their multicultural identity experiences in an open, accessible and concrete manner; in this section, the role of the interviewer was completely passive as the participant constructed and communicated their story as they wished. The narrative structure also captured participants' well-being through their story's coherence. The second half of the interview consisted of questions specifically inquiring into their identity configuration experience. Any potential bias to the narrative section was avoided by the participant-directed format of the narrative. Moreover, there were no carry-over or order effects from the identity configuration section to the narrative since the interview was presented as having two different sections, and since the narrative section consistently preceded the multicultural identity configuration questions.

#### The cultural identification narrative

McAdams' (1989, 1992; Baerger and McAdams, 1999) life narrative procedure was adapted for the interview process, where interviewees were asked to tell the story of growing up with their different cultural groups, and their cultural identity development rather than their entire life story. Participants were instructed to think of their multicultural experience as a story, with characters, settings, high and low points, and to tell their story using chapters as a framework, from where they felt it began to their present day (see Appendix). In line with the original procedure (e.g., McAdams, 1985, 1989, 1992), the instructions were open for the participants to freely organize their stories as they wished. No suggestion was made for the participants to prioritize any of their cultural groups, or that they configure their identities in a certain way. The stories ranged from two to six chapters in length; fifteen participants organized their stories chronologically, four split each culture into its own chapter, and three participants had a hybrid of the two structures. For the chronological and hybrid structures, each chapter that took place in childhood was recoded as a childhood chapter, while those that took place in adolescence or adulthood were recoded as adolescence and adulthood chapters, respectively.

#### Cultural identity configuration questions

After participants completed their cultural identification narrative, they were asked specific, open-ended questions about the relationships between their cultural identities (see Appendix). These questions were designed to capture participants' categorization, compartmentalization, and integration identity configurations based on the CDSMII. Examples of questions for each configuration include: “Do you feel a greater preference for one culture over the other(s)?” (categorization); “Do you feel very different from one cultural context to another?” and “Do you prefer to consider each cultural identity as being very distinct and separate from each other?” (compartmentalization); “Do you see a lot of common ground between your cultural identities?” and “Do you feel that you can identify yourself, for example, as a ‘global citizen,’ or ‘Asian,’ or some other broad category that would include the different cultural identities?” (integration).

### Data preparation

Following Baerger and McAdams (1999), the narratives were rated for their coherence as an indicator of well-being. Narrative coherence denotes how well the narrative holds together in terms of its adherence to a traditional cause-effect, or event-response pattern on the four dimensions of orientation, structure, affect, and integration. The degree to which each of these four components was found in the narrative was rated with a 7-point Likert scale ranging from 1 (Very low) to 7 (Very high), and was determined by how intense, frequent and embedded each component was within the story. This scale was used to rate each chapter in one's narrative, as well as the narrative as a whole. The scores for each of the four coherence components are added to create the overall score of narrative coherence, as per the procedure investigated by Baerger and McAdams (1999). There were two coders for the narratives. Inter-rater reliability was calculated with 50% of the narratives, which was coded by both independent raters; Pearson correlations between the raters demonstrated satisfactory inter-rater reliability for the ratings of narrative orientation (*r* = 0.82, *p* = 0.000), structure (*r* = 0.94, *p* = 0.000), affect (*r* = 0.80, *p* = 0.001), and integration (*r* = 0.90, *p* = 0.000). Table 1 consists of excerpts of narrative sections that were coded as representing low (coded as 1–2 on the 7-point scale), moderate (coded as 3–5), and high coherence (coded as 6–7).

**Table 1:** **Narrative excerpts from the current study illustrating low to high ratings of coherence using the 1–7 Likert scale from Baerger and McAdams (1999)**.

**Coherence level**	**Excerpts**
Low coherence (coded between 1–2)	“Childhood, grades, languages, I guess was my big thing. I guess if I count French, I know five languages. English, Mandarin, Cantonese, and this language really close to Taiwanese its Teochew-nese. … So languages is part ‘cause there's a school on Saturdays for that, for Mandarin. ‘Cause Cantonese I speak at home and Mandarin I learned at school ever since I was four so at the same time I learned piano. So I went to school ever since then and I watched TV dramas whatever to practice my Mandarin. That's where most of my Mandarin comes from. My slang in Mandarin is better than the ones who live there. So I would talk to my friends, like, say some slang or whatever and they would have no, it's like, “Oh really!” or “Wow you know that?” “Have you ever been there?” So I would go to school and they're like “Wow! You go to school on Saturday?” and I was like “Yeah!” I felt cool you know and all that … I did really well in school when I was in elementary school. Now thinking back, I say I should've played around more, like, why get good grades in elementary school, that doesn't count! University counts, really, truly. That was my childhood. Oh there's an incident in grade two and so I live here and then there's a cul de sac [dead end street] right across from my house. And then, me and my sister would do biking there and this guy is from the, goes to the same elementary school, in the same class even, so that summer we would bike around. We wouldn't really talk, like, he's white and he has an older sister, he's fine. Anyways, we would ride our bikes whatever and he would try to do a wheelie and we would try to do it with him, but we wouldn't really talk. He'd be like “hey” and then start biking, ride around, ride here, ride there, follow him. And we would do that for like I don't know fifty minutes, half an hour maybe once a week or something. And then it was in grade two, so we were, like, seven. And then at the end of that school year, he bought Spice Girl bubble gum. Bubble gum that had Spice Girls stickers. And he gave it to every single girl except for me. I was sad. I don't know what, he said “because you're mean.” I was like, “I'm the nicest person that I know!”
Moderate coherence (coded as 3–5)	“So now, it's like, being ok with my culture, realizing that I am always learning new stuff about my own cultures. How I relate to them, and that, like I was saying about language, also it's open to me to explore my culture more. It is open to me to learn Chinese, and to try and tap into that, and to try and communicate with my family. The thing that has discouraged me for a long time is because there are many, many dialects within the Chinese language. Not least of all Cantonese versus Mandarin, and the subdivisions. So my family speaks Cantonese which is the more difficult of the languages to learn, and my great-grandmother, who I want desperately to speak to, speaks Toisan, which is, like, even more difficult and obscure. And, like, who am I going to get to teach me that? So I think if I do anything, I will try and learn some basic Cantonese, and then if I can get, find somebody else, or go home and try and hack it out with them. Figure out something. But, like, it's daunting because I am actually, like, extremely scared of failure in that regard because, like, it has so much now, attached for so long, I've wanted to learn. Ok I'll put it off. And in my head I am always like, “Oh I'll do a summer course, I'll do this.” Since high school I was planning on doing it and they didn't offer Chinese at my school. I would've taken it. But they would've only offered Mandarin anyway. Which my brother tried, ‘cause I am sure he was struggling with his own things, too. It's funny that we haven't talked about it that much. And he took Chinese at his school because he went to a school that was in like, closer to Chinatown. So lots of Chinese people. And so he was like the white dude in the class. But that was the problem because everybody already spoke, they just, none of them wrote. They were all, like, first generation [Canadian born] you know their parents would read to them but they never went to school for Chinese. So they were learning to write, so he [brother] learned to write but he couldn't say anything. He was just reading, he knew what it meant. So yeah my grandmother is telling me learn Mandarin, ‘cause to her she is just like, she is still “Fit in or do something pragmatic, learn Mandarin because that is the language of business and money.” She is always like, “Go into business or get a pharmacy. You're a smart woman, you know those pharmacists ain't dumb.” So she is telling me this, and I am like, “But I want to learn to speak to you.” I don't care, plus lots of people speak Cantonese, everybody in Chinatown here speaks Cantonese. And all the best movies are in Cantonese.”
High coherence (coded as 6–7)	“There comes this time where I got promoted to the student council … And the job is to enforce the school rules, you know check girls skirt length and so on and so forth. But we also had to check for absences, so we had this little card we had to fill in and we knew we had to tell each teacher, “we know who is there in the morning” … Ok, so I just told the teacher and she was like, “Oh, this is not the first time because I know they have been missing also,” and so she told the powers that be at school, and they [the students] got into trouble. But they [the students] all came to see me, and that was a big revelation of my life, because that is where the line dropped: “Oh it's because you are Chinese that you did that, if it was your Chinese friend you would not have done that.” So I was like: “What the heck?” … I was so angry. I was just mad, I was frothing because it was the first time that anyone had told me that I had made any preference … It's the first time that anyone had told me that, because with all the weight that is implied with “Oh, you're smart because you are Chinese, you're rich because you are Chinese” and so on and so forth, “Oh, you did that because you are Chinese, so you can oppress other people” … and I was so mad afterwards, I just went to my best friend, who was of Indian origin, but didn't feel Indian in the least. So I went to her and was like, “You know what, that is the first time that anyone has told me that in such a way and I was really mad.” She was like, “well, can you tell me why?” I was like, “You know, I don't feel Chinese. It's just like everyone has been telling me ever since I was very little, “Oh you are Chinese, so you must be this,” or “You are Chinese so you must be that.” No, I don't like math, I don't like science, I don't like economics. I like art, I like literature, I like languages. Everyone is telling me something is wrong with me because I am Chinese I am not doing the things that I am supposed to do … You know, it's like I just don't feel like I am that person. This is not who I am.” She was like “fine, so who are you?” I was like, “Hmmm good question, who am I?” So that bugged me. That was the start of what I would say, you know questioning time. And it really, really, really, like, woke me up.”

Each instance of categorization, compartmentalization, and integration that emerged throughout each interview was respectively coded in accordance with the definitions provided in the CDMSII. In order to determine which multicultural identity configuration was dominant for each individual, the degree to which each of these statements represented categorization, compartmentalization, and integration was respectively rated on a scale of 1 (not at all) to 7 (completely). The mean of each configuration was calculated and the greatest mean represented the participants' dominant cultural identity configuration. From this procedure, 7 participants reported predominantly categorized identification (with the Canadian identity being the dominant cultural identity for all categorized participants), 3 reported predominantly compartmentalized identification, and 12 reported predominantly integrated identification. There were no tie ratings among each participant's ratings. The average difference between each identity configuration rating of 1.67, (*SD* = 0.85), and a range of 0.09–4.72. This classification enabled us to compare each cultural identity configuration type for the outcome of narrative coherence. Pearson correlations between the two independent raters demonstrated highly satisfactory inter-rater reliability between the mean ratings for categorization (*r* = 0.97, *p* < 0.01), compartmentalization (*r* = 0.92, *p* < 0.01), and integration (*r* = 0.97, *p* < 0.01) based on 72% of the interviews.

To avoid confusing the identity integration construct provided by the CDMSII with McAdams' concept of integration as one of the four indicators of narrative coherence, we employ the term “narrative integration” to refer to the integration denoted in participants' narratives, and “cultural identity integration” to refer to the integration stage of the CDMSII.

## Results

### Overview of the analyses

We first present how individuals spoke about their own identity experiences in light of the configurations postulated in Amiot et al. (2007) model, including illustrative direct citations. Preliminary analyses are then presented, including Cochran's *Q*-tests that were performed to examine how people's identity configurations evolved across the chapters of their narratives. Following this, analyses testing our hypotheses were conducted. We hypothesized that each identity configuration would be associated with different levels of narrative coherence, and that those with integrated cultural identities would report higher narrative coherence than compartmentalized individuals, and that categorized would report lower narrative coherence than compartmentalized individuals. To test these predictions, correlations were conducted between the means of the different identity configurations for the entire sample and the dimensions of narrative coherence, followed by ANOVAs comparing individuals who are dominant in either categorization, compartmentalization, and integration on the different dimensions of narrative coherence.

### Illustrative quotes

Participants spontaneously talked about the categorized, compartmentalized, and integrated cultural identity configurations in their own words as they recounted their life stories, and also mentioned these configurations in response to the specific cultural identity configuration questions. A selection of illustrative quotes are presented in this section:

#### Categorization

Categorized participants referred to the experience of excluding one identity while favoring another. Illustrating this strategy, K. Y., a Chinese–Canadian participant, conveyed her identification by stating, “I still consider myself Canadian, not Asian. I don't like my Asian culture.” demonstrating a preference for her Canadian identity while excluding her Asian heritage culture. A. A., a half Italian, half Chinese–Guyanese Canadian said “I'm Canadian because I'm neither Italian nor Chinese, just by heritage, and the Guyanese part doesn't really come in because that's not me, that's my mother.” Her experience illustrates her predominant Canadian identity as well as her representation of her heritage cultures as part of her lineage, but not part of her self-concept.

#### Compartmentalization

Illustrating compartmentalization, L. V., a half Israeli-Jewish, half Irish Canadian, said “It's not that one [identity] is inferior to the other, it's just separate.” Here, L. V. shows that both identities are equally part of her self-concept, but that she sees them as separate from each other. J. G., who is half Italian and half Irish–British Canadian, describes her experience of only identifying with each culture in their respective contexts: “I have different cultural experiences within my being like half there, half there, so that I can bounce between two cultures, three cultures, four cultures … I feel different [in different cultural contexts] because it was always one or the other. It was never both at the same time.”

#### Integration

For integration, K. M., who is half Chinese, half Euro–Canadian, illustrated the experience of seeing similarities between her identities by stating, “Within myself, I have these two identities, and so I can find common things between them.” K. C., who comes from Cantonese–Chinese parents, and was born and raised in Montreal, spoke about how he resolves the differences between his Chinese and Canadian traditions, and how he sees these differences as mutually beneficial for him: “So what I like out of the Chinese tradition is a lot of respect and seeking to understand tradition, which I think is very important, but what I get out of my Western upbringing is the questioning at the same time. And so it enriches each other because every time I see an established tradition … I'll discuss and be very respectful, but at the same time as I'm discussing and being respectful, I'm not just staring at it blank-faced and just taking it on, which would be totally destructive of tradition in the first place ‘cause you lose the sense of what it is.”

M. G., who is half Lebanese, half French, born in the United States, and moved to Montreal during his childhood, demonstrated how he adapts to his cultural contexts while still retaining his interconnected, simultaneously present identities: “I am now like a Swiss army knife. Multiple tools, and you can pretty much deal with anything, if I need a screw driver, it's in there. So if I need to be American, I'm in here … Certainly when I am talking to a Lebanese person, and I have multiple cultures behind me, I do feel a bit more Lebanese at that specific time, just at that specific time. But that doesn't mean that the rest of it is gone. No. You are going to use the rest of that to, you know, make the conversation more interesting. Soften the mood, make them feel more welcome.”

### Preliminary analyses

When averaging the total number of instances that these configurations were mentioned in each participant's interview, descriptive analyses showed that the frequency of reported categorization ranged from 0 to 20 instances across the interviews (*M* = 7.41, *SD* = 6.12); the frequency of reported compartmentalization strategies ranged from 1 to 24 occurrences (*M* = 7.73, *SD* = 6.32), while the frequency of reported integration strategies ranged from 2 to 29 instances (*M* = 13, *SD* = 8.08). These findings suggest that participants varied in the extent to which they each mentioned these identity configurations.

Given that the developmental literature (e.g., Phinney, 1989; Harter et al., 1997) and the CDSMII propose that the development of these identity configurations is dynamic, analyses were conducted to verify if the spontaneous reporting of one's configurations would vary over the course of the life story, as retrospectively narrated by the participants. To track how the evolution of people's identification experiences were constructed retrospectively through the narration of their own life stories, the proportions of the different identity configurations were examined across the childhood, adolescence and adulthood chapters of each narrative for the eighteen participants who organized their narratives chronologically. Whether a chapter was designated as childhood, adolescence, or adulthood was determined by the participants themselves since they themselves clearly named and described the content of each of their chapters. For example, participants named their chapters “childhood,” “elementary school,” “teenage years,” “high school,” “adulthood,” “university,” and “present day.” Cochrane's *Q*-tests demonstrated a significant difference between chapters in terms of the frequency with which instances of compartmentalization were mentioned [*Q*_(2)_ = 7.00, *p* = 0.03]: The percentage of participants who reported instances of compartmentalization in their childhood chapter was 11%, whereas 33% of participants reported compartmentalization strategies in the adolescence chapters, and 6% reported compartmentalization in the adulthood chapters. Follow-up McNemar tests were conducted to determine which chapter significantly differed from the other in terms of compartmentalization; these tests revealed that the decrease from the adolescence to adulthood chapter was marginal (*p* = 0.06), the other percentages did not significantly differ. There was a significant difference across the chapters for identity integration [*Q*_(3)_ = 9.00, *p* = 0.01]. In both childhood and adolescence chapters, 6% of participants reported integration strategies. Follow-up McNemar tests revealed that this percentage significantly increased from the adolescence to the adulthood chapter (39%; *p* = 0.03). The reported proportion of the categorization configuration was not statistically different across chapters [*Q*_(1)_ = 2.00, *p* = 0.37]; the percentage of participants who reported categorization strategies tended to increase from the childhood (28%) to adolescence chapters (44%) and then remained stable in the adulthood chapter (44%) but these percentages did not differ significantly. These findings show that participants' recounting of their identity configurations evolved through their life story in a logical manner. The patterns observed are in accordance with the developmental literature (e.g., Harter and Monsour, 1992; Harter et al., 1997), which reported the emergence of multiple identification issues and conflicts in adolescence, and resolution of these conflicts by adulthood.

### Correlations among the constructs

Correlations were conducted between the means of each identity configuration across the entire sample and the four narrative coherence indicators. As can be seen in Table 2, significant correlations between the means of each identity configuration and the overall narrative coherence ratings demonstrated no significant correlation between overall narrative coherence and categorization (*r* = 0.004, *p* = 0.99), while overall narrative coherence was significantly and negatively related to compartmentalization (*r* = −0.66, *p* = 0.001), and positively related to cultural identity integration (*r* = 0.53, *p* = 0.01). The relationships between the different configurations and each of the four dimensions of narrative coherence were further examined, revealing a similar pattern across the configurations (see Table 2); categorization was not significantly related to any of the four dimensions. Compartmentalization was related negatively to the four coherence indicators: orientation, structure, affect, and narrative integration. As expected, integration was positively related to structure, affect, and narrative integration. A similar pattern was observed where identity integration was positively related to the orientation dimension of narrative coherence, but this relationship was not significant.

**Table 2:** **Intercorrelations between the multicultural identity configurations and narrative coherence indicators**.

**Variables**		***M***	***SD***	**1**	**2**	**3**	**4**	**5**	**6**	**7**
1.	Categorization	2.22	1.15	–						
2.	Compartmentalization	2.22	0.95	−0.19	–					
3.	Integration	3.36	1.34	−0.69^**^	−0.53^*^	–				
4.	Overall narrative coherence	4.68	1.27	−0.00	−0.66^**^	0.53^*^	–			
5.	Narrative orientation	5.09	1.23	0.18	−0.60^**^	0.32	0.80^**^	–		
6.	Narrative structure	4.18	1.53	−0.06	−0.49^*^	0.47^*^	0.87^**^	0.60^**^	–	
7.	Narrative affect	5.14	1.36	0.09	−0.60^**^	0.47^*^	0.81^**^	0.62^**^	0.52^*^	–
8.	Narrative integration	4.32	1.81	−0.10	−0.58^**^	0.54^**^	0.93^**^	0.61^**^	0.80^**^	0.68^**^

Note: *N* = 22,

* p < 0.05 level,

** *p < 0.01 (2-tailed)*.

### Differences in narrative coherence between the cultural identity configurations

**Figure 1:**
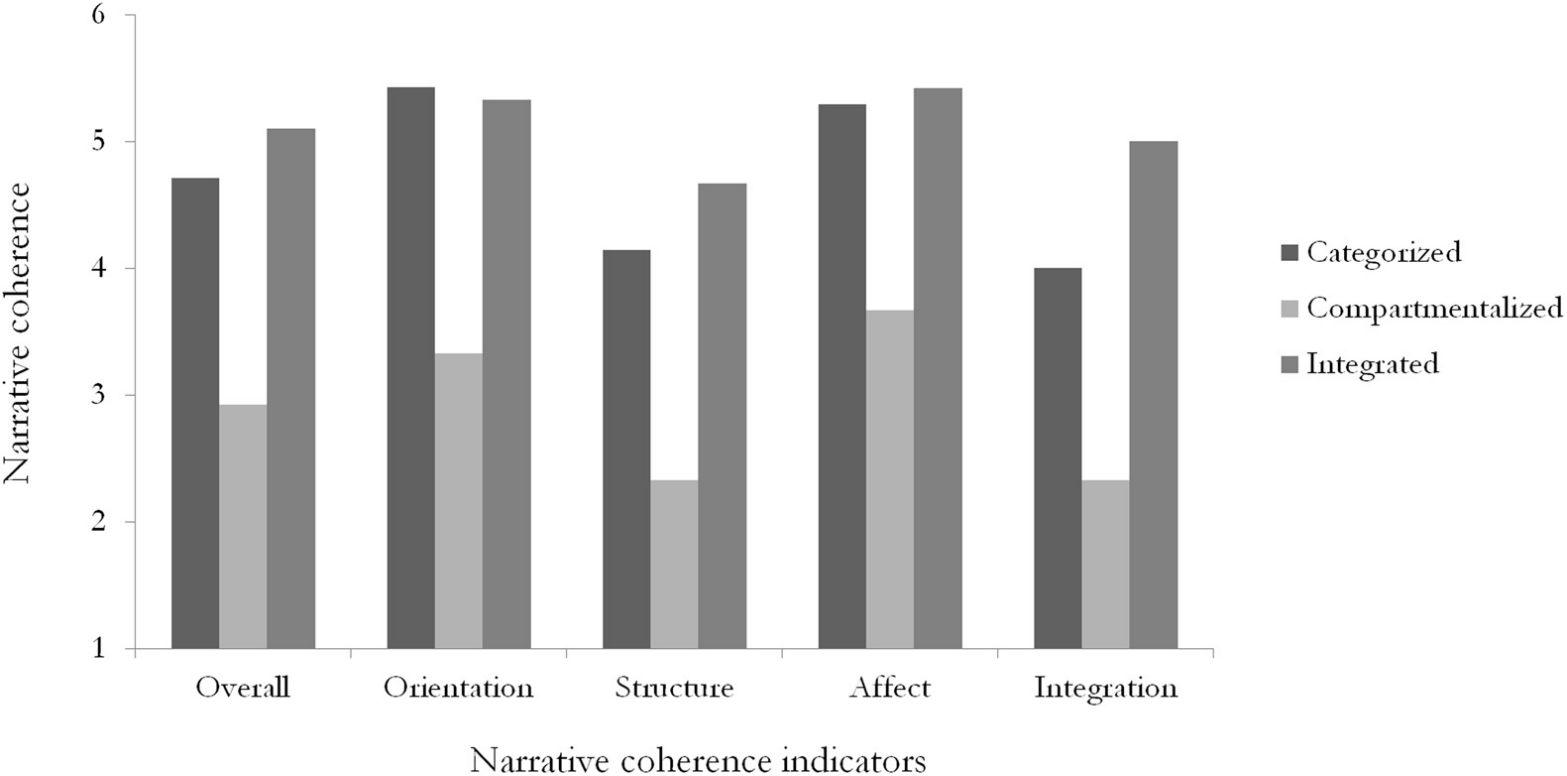
**ANOVA results of multicultural identity configurations to the narrative coherence indicators**.

Following the classification of participants into their dominant identity configuration, we expected that those with integrated cultural identities would display the greatest overall narrative coherence and higher scores on all four coherence dimensions compared to those with compartmentalized and categorized cultural identities. An initial ANOVA investigating the overall narrative coherence of each identity configuration showed a significant main effect of cultural identity configuration on overall narrative coherence [*F*_(2, 19)_ = 4.85, *p* = 0.02]. *Post-hoc* Tukey's HSD tests revealed that integrated participants (*M* = 5.10, *SD* = 1.15) had significantly more coherent narratives overall than the compartmentalized participants (*M* = 2.92, *SD* = 0.95, *p* = 0.02); categorized participants (*M* = 4.71, *SD* = 1.01) had marginally more coherent narratives than those with compartmentalized identities (*p* = 0.067). There was no significant difference between those with integrated and categorized cultural identities (*p* = 0.74). Subsequent univariate ANOVAs were conducted for each dimension of narrative coherence, and this general pattern of findings was also found across the four indicators of orientation, structure, affect and integration (see Figure 1).

#### Orientation

There was a significant main effect of cultural identity configuration to the orientation dimension of narrative coherence [*F*_(2, 19)_ = 4.86, *p* = 0.02, η^2^ = 0.34]. Pairwise comparison follow-up analyses revealed that integrated (*p* = 0.008) and categorized participants (*p* = 0.009) had significantly higher orientation ratings than the compartmentalized participants; There was no significant difference between those with integrated and categorized cultural identities (see Figure 1).

#### Structure

Results showed a marginal main effect of cultural identity integration to narrative structure [*F*_(2, 19)_ = 3.43, *p* = 0.05, η^2^ = 0.27]. Pairwise comparisons demonstrated that those with integrated cultural identities had significantly higher structure ratings than those with compartmentalized cultural identities (*p* = 0.02). Categorized participants had marginally higher structure ratings (*p* = 0.073) than compartmentalized participants (see Figure 1). There was no significant difference between culturally integrated and categorized individuals.

#### Affect

There was a non-significant main effect of cultural identity configuration to narrative affect [*F*_(2, 19)_ = 2.32, *p* = 0.13, η^2^ = 0.20]. The results displayed a similar pattern to the structure dimension; pairwise comparison follow-up analyses showed that participants with integrated cultural identities had significantly higher narrative affect than compartmentalized participants (*p* = 0.047). Categorized participants had marginally higher narrative affect (*p* = 0.082) than participants with compartmentalized cultural identities. Those with categorized and integrated cultural identities did not differ significantly on the affect dimension (see Figure 1).

#### Integration

A marginal main effect of cultural identity configuration to narrative integration was obtained [*F*_(2, 19)_ = 3.40, *p* = 0.055, η^2^ = 0.26]. Pairwise comparisons revealed that individuals with integrated cultural identities had significantly higher narrative integration (*p* = 0.02) than individuals with compartmentalized cultural identities There was no significant difference between categorized and compartmentalized participants, nor between participants with categorized or integrated cultural identities (see Figure 1).

These findings suggest that categorized and integrated individuals' narratives presented greater coherence compared to those of compartmentalized individuals. This was found for all domains of narrative coherence for those with integrated cultural identities; for categorized individuals, this was found specifically for orientation, with marginal effects for the structure and affect domains. These findings confirm our hypothesis that integration predicted greater narrative coherence than compartmentalization. We also expected that categorization would be associated with lower narrative coherence, but our findings do not support this prediction. This unexpected finding will be addressed in the “Discussion” section.

## Discussion

These findings provide rich insight into the identity configurations and the life experiences of multicultural individuals. Participants' spontaneous communication of their own multicultural identification reflected the identity configurations proposed in the CDSMII. Furthermore, the positive relationship between identity integration and narrative coherence, especially compared to the other configurations, confirms the proposed link between identity integration and well-being (Amiot et al., 2007).

The results comparing each cultural identity configuration on their narrative coherence ratings confirm the hypothesis that the integrated multicultural identity configuration is associated with greater well-being compared to compartmentalization, as indicated by how coherently participants convey their own multicultural story. These findings suggest that the coherence within the self that is characteristic of multicultural identity integration is associated with the ability to frame the context of one's narrative, to convey one's story in a logical order, and to create a sense of overall meaning and resolution in one's narratives. In separating and contextualizing their cultural identities, compartmentalized individuals may have a more fragmented view of the self in relation to their cultures. This compartmentalized identity configuration may then inhibit them from concretely setting the stage for their cultural narrative, from providing a logical sequence to their story, from having a clear or present emotional evaluation of the events in their narratives, or from deriving meaning or resolution from their situation. These findings for identity integration and compartmentalization to narrative coherence are also consistent with the previous work on cultural identity integration and well-being (e.g., Downie et al., 2004; Cheng et al., 2008; Carpentier and de la Sablonnière, 2013).

We originally hypothesized that categorized multicultural individuals would display lower narrative coherence than compartmentalized individuals, such that identifying with one identity over the others would be associated with less coherent narratives. The findings for categorization showed that categorized individuals performed better than compartmentalized individuals for the indicators of narrative coherence, sometimes comparably to those with integrated cultural identities. These results were unexpected. There are several explanations for these findings. First, previous research demonstrated that identifying with one cultural group over others did not predict well-being while identifying with multiple cultural groups did relate to more well-being (e.g., Berry et al., 2006; Nguyen and Benet-Martínez, 2010, 2013; Carpentier and de la Sablonnière, 2013). At the same time, the majority of the literature on bicultural or multicultural individuals thus far has been conducted mostly among first generation immigrants relative to second or third generation individuals, or those with a “mixed” cultural heritage. The sample of the present study was composed mainly of second generation and “mixed” heritage individuals, and the categorized participants identified predominantly with the mainstream cultural group (i.e., being Canadian). In the present research, there may have been an effect of generation status: identifying primarily with the majority cultural group may not compromise well-being among this particular population, given that the second generation grew up in the context of the majority group, and have therefore identified with the group and context that they are most familiar with.

Another possible explanation is the nature of the identity that is endorsed in a categorized configuration. The categorized participants in the current study all identified predominantly as Canadian. Schwartz' values survey (1994) demonstrated that Canada valued egalitarianism; these values can be observed in Canada's Constitution and Charter of Rights and Freedoms (Government of Canada, 1982), which endorse equal rights, tolerance, and multiculturalism. In this vein, Uskul et al. (2007) found that Chinese Canadians' endorsement of their Canadian identity was positively correlated with their personal openness to date interculturally. It is possible that the categorized participants, who all identified primarily as Canadians, perceived Canada to be an open and egalitarian context, thus engendering a sense of inclusiveness and tolerance, which, in turn, may have affected their well-being positively, as captured by their narrative coherence.

Third, perhaps the motivations and social context behind one's alignment with one culture are at play. It is possible that categorization could predict coherence if the predominant cultural identity was chosen agentically rather than induced from group pressure or lack of opportunities (e.g., Chirkov et al., 2005; McAdams et al., 2006). The intergroup relations literature has examined the difference between assigned vs. chosen social identities (e.g., Turner et al., 1984; Deaux, 1991; Perreault and Bourhis, 1999). In their experiment on the effects of chosen versus assigned group identities in the context of intergroup competition, Turner et al. (1984) demonstrated that choosing one's own group lead to greater collective self-esteem than being assigned to a group. These findings suggest that choosing one's cultural identification may also be linked to well-being; this process may have been at work for the categorized participants in this study, who reported greater well-being. To clarify this finding, future studies will need to directly assess and account for the extent to which individuals feel that they are guided by their own personal choice when identifying predominantly with one cultural group to the exclusion of others.

In terms of potential implications for a clinical context, these findings suggest that some identity configurations may be more adaptive than others. At the same time, further evidence is needed to paint a more complete picture of multiculturals' identity processes and their mental health. Therefore, before drawing conclusions about whether one cultural identity configuration should be preferred or recommended over another, future research using a variety of methodologies—including qualitative and quantitative approaches, various measures of identity configuration, and established well-being and mental health instruments—should be conducted to gain a greater understanding of the relationship between multicultural identity configurations and well-being outcomes. Moreover, attention needs to be given to the fundamental contribution of various social factors in shaping how multiculturals configure their identities. Future research will be conducted to deepen our understanding of how the social context predicts multicultural identity configuration, as well as how these configurations, in turn, predict a range of well-being outcomes.

The narrative approach typically employed for research purposes could also be beneficial for use in clinical practice with multicultural people. Its previous use in measuring well-being, as well as in clinical research has demonstrated its effectiveness in capturing the process of well-being for the participating person (e.g., Baerger and McAdams, 1999; Roe, 2001; Lysaker and Lysaker, 2002; Adler et al., 2007). In addition to gaining greater understanding of the client's well-being through the evaluations of coherence, the narrative method provides a rich account of each individual's unique and subjective appreciation of their life trajectory, history, social context, and how they arrived at their current state (McAdams, 1985). These insights into multicultural individuals' history and identity could prove a useful initial step for care practitioners in building a rapport with, and profile of, their clients.

While the life story procedure has been implemented with multicultural populations in prior research (e.g., Bougie et al., 2011; de la Sablonnière et al., 2011), it is possible that the current format of creating a story using chapters and analyzing its coherence through orientation, structure, affect and integration could stand to be more culturally sensitive or culturally specific. It would therefore be valuable for future research to consider examining different narrative styles found in different cultural groups' storytelling traditions in order to adapt this method to include the potential cultural variations in making a well-constructed, coherent life narrative (Brewer, 1985; Shweder and Much, 1987; Pavlenko, 2002; McAdams, 2006).

One of the limitations of the present study was the use of a single indicator for well-being. Future qualitative inquiries could conduct a multicultural narrative interview and include established and/or conventional clinical interview schedules (e.g., Brugha et al., 1999; Subramaniam et al., 2006) as additional indicators of mental health. Future quantitative research will also be conducted on a larger scale to examine the relationship between the multicultural identity configurations to multiple indicators of subjective and psychological well-being.

The qualitative nature of the present study resulted in a sample size of twenty two participants; another limitation of the present study is the low representation of compartmentalized individuals in our sample (*n* = 3); while there were few compartmentalized participants, the inter-ratings concluded the same configuration assignment, and results from the ANOVAs comparing members from each configuration are consistent with the correlation results examining compartmentalization for the entire sample. Nevertheless, greater representation of compartmentalized people is warranted; future quantitative research, therefore, will test a larger sample of multicultural individuals to better represent each configuration.

The current research sought to qualitatively examine multiculturals' identity configuration process and well-being as indicated by their narrative coherence. The present study provides evidence that integrating one's multiple cultural identities within the self predicts greater well-being than compartmentalizing one's identities; adopting one identity over the others seems to be associated positively with measures of coherence, pointing to a need for a more nuanced understanding of this particular configuration. Future large-scale studies will continue to examine the relationship between a variety of well-being measures and the cultural identity configurations using a scale that is being developed for this purpose of quantitatively assessing these configurations. Moreover, these studies will focus on the social context of multicultural individuals, and how a variety of social factors can influence how one configures one's multiple cultural identities within the self. These forthcoming investigations will complement the present study, and continue to build on the body of work that seeks to better grasp the experiences of multicultural individuals.

### Conflict of interest statement

The authors declare that the research was conducted in the absence of any commercial or financial relationships that could be construed as a potential conflict of interest.

## Appendix: interview questions

### Introduction questions: what are your cultural groups?

- Please describe each group.
- How long have you been a member of each group?

### Cultural narrative procedure

#### Introduction

This is an interview about the story of your cultural identification. In this interview, I'll ask you to play the role of a storyteller about your own life with your cultures. I'd like you to construct the story of your own past and present in relation to your cultures.

In telling us about your own multicultural story, you do not need to tell us everything that has ever happened to you. A story is selective. You may focus on a few key events or a few key themes that you believe to be important. Think about things in your own life which say something significant about your cultural identification(s) and how your cultural identification has developed up to this point in the present. Your story should tell me how you are similar to other people as well as how you are unique in terms of your cultural identification. I will guide you through the interview so that we can finish in good time. Do you have questions?

#### Chapters

I would like you to begin by thinking about your cultural identification as a story. All stories have characters, scenes, scenarios, and so forth. All stories have high points and low points, good times and bad times, heroes and villains, and so on. A long story may even have chapters. In this interview, I would like you to think about the story of your cultural identities as having at least a few different chapters.

In this section of the interview, I would like you to describe for me each of the main chapters of your cultural identification story, from where you see it beginning until now. You may have as many or as few chapters as you like, but I would suggest dividing your story into at least 2 or 3 chapters and at most about 7. I would like you to give each chapter a name, and describe briefly the overall contents in each chapter. By overall content I mean the key theme or event that happened to you in each chapter. As a storyteller here, think of yourself as giving a brief summary for each chapter.

You may need time to think about what the main chapters in your story would be. If you want, you can write them down. When you're ready, we will go through each of your main chapters (so that you can tell me a little bit more about them).

### Multicultural identity integration questions

- Categorization: Do you feel a greater preference for one culture over the other(s)?
- Compartmentalization: Do you prefer to consider each cultural identity as being very distinct and separate from each other?
- Compartmentalization: Do you feel very different from one cultural context to another? (or “depending on the cultural context”)
- Integration: Do you see a lot of common ground between your cultural identities?
- Integration: Do you feel that you can identify yourself as a “global citizen,” or “Asian,” “North American,” or some other broad category that would include the different cultural identities?
